# Simultaneous Biarticular Growth Modulation for Ipsilateral Concomitant Valgus Deformities of the Knee and Ankle: Short-Term Results of a Case Series

**DOI:** 10.3390/children13050675

**Published:** 2026-05-14

**Authors:** Robert Hennings, Thibaut Hiegel, Daniel Gräfe, Christoph-Eckhard Heyde, Eckehard Schumann

**Affiliations:** 1 Department of Orthopaedic Surgery, Traumatology and Plastic Surgery, University Hospital Leipzig, Liebigstrasse 20, 04103 Leipzig, Germany; thibaut.hiegel@medizin.uni-leipzig.de (T.H.); christoph-eckhard.heyde@medizin.uni-leipzig.de (C.-E.H.); eckehard.schumann@medizin.uni-leipzig.de (E.S.); 2 Department of Pediatric Radiology, University Hospital Leipzig, Liebigstrasse 20, 04103 Leipzig, Germany; daniel.graefe@medizin.uni-leipzig.de

**Keywords:** Hemiepiphysiodesis, eight-Plate™, EPI Plate, tension band plate, genu valgum, ankle valgum, growth modulation, guided growth

## Abstract

**Highlights:**

To the best of our knowledge, this is the first analysis of a larger case series of children with concomitant knee and ankle valgus deformities (CKAVD) treated with biarticular hemiepiphysiodesis using a tension plate. For these children, simultaneous ipsilateral biarticular hemiepiphysiodesis (HED) seems to be an efficient and low-complication approach to correcting knee and ankle valgus deformities. With careful patient selection and precise surgical planning, this method achieves correction within ranges previously reported in the literature for monoarticular HED. The possibility of a two-stage treat-ment termination should be transparently discussed with patients and their guardians before initiating biarticular HED. The need for this approach should be evaluated throughout therapy via periodic follow-up examinations.

**What are the main findings?**

- Simultaneous biarticular HED achieved physiological alignment in nearly all patients within a mean modulation period of 14.3 months.
- A two-stage termination strategy seems to prevent overcorrection in cases where joint correction rates of the knee and ankle joints diverged.

**What are the implications of the main findings?**

- For children with symptomatic CKAVD, simultaneous ipsilateral biarticular hemiepiphysiodesis (HED) may be considered as a treatment option, presented as feasible with promising short-term outcomes and a low complication rate.
- The possibility of a two-stage treatment termination by hardware removal should be considered.

**Abstract:**

Background/Objectives: Valgus deformities of the knee and ankle are frequently diagnosed during adolescence. Hemiepiphysiodesis (HED) using the tension-plate technique has become the standard approach for guided growth in both joints. Notably, valgus deformities may simultaneously affect both joints. While substantial data supports monoarticular guided growth, evidence for ipsilateral biarticular growth modulation remains limited. The aim of this case series was to evaluate the feasibility, safety and short-term radiographic outcome of simultaneous ipsilateral biarticular HED for concomitant valgus deformities in the knee and ankle (CKAVD). Methods: This retrospective monocentric observational study included 21 legs from 21 consecutive children treated with simultaneous ipsilateral biarticular HED for CKAVD between 2013 and 2022. The initiation and termination of growth modulation were based on clinical (intermalleolar distance, pain in both joints) and radiological parameters. Standing whole-leg coronal plain radiographs were evaluated at the start of modulation and after complete hardware removal, assessing the anatomical femorotibial angle (aFTA), anatomical lateral distal femoral angle (aLDFA), medial proximal tibial angle (MPTA), and lateral distal tibial angle (LDTA). Results: The mean age at implantation was 11.5 years (SD 1.08); females (n = 7) were younger than males (n = 14, *p* < 0.05). The mean duration of growth modulation was 14.3 months (SD 4.3). The intermalleolar distance improved by an average of 10 cm (SD 4.4). The aFTA improved by an average of 6.9 degrees (SD 2.6), the aLDFA by 6.7 degrees (SD 2.7), the MPTA by 7.2 degrees (SD 2.3), and the LDTA by 7.1 degrees (SD 3.0). Two-stage hardware removal was required in 6 legs (29%). In one case, a relapse of knee valgus occurred. Conclusions: Comprehensive evaluation of all major joints seems to be crucial during the diagnostic workup for coronal plane deformities of the lower extremity. For children with ipsilateral symptomatic CKAVD, simultaneous biarticular HED may be considered as a feasible treatment approach, demonstrating promising short-term outcomes and a low observed complication rate.

## 1. Introduction

Coronal plane deformities of the lower extremities are commonly encountered in skeletally immature patients [1,2,3,4]. A comprehensive medical history is essential to elucidate the underlying etiology, with obesity and idiopathic causes being the most common [1]. Particular attention should be paid to asymmetric deformities, which are often associated with congenital musculoskeletal or metabolic disorders, including multiple hereditary exostoses (MHE), bone dysplasia, myelodysplasia, tumors, or fibular deficits [5]. Additionally, post-traumatic, post-infectious, or immunogenic etiologies should be considered [5]. In the cases of ankle valgus, the differential diagnoses should also include neuromuscular conditions such as myelomeningocele, poliomyelitis, cerebral palsy, or MHE [4,6].

Pathogenetically, secondary deformities commonly result from unilateral physeal disturbances, leading to asymmetric growth and altered load distribution with subsequent axial malalignment. In MHE, relative hypoplasia of the lateral tibial epiphysis promotes disproportionate medial growth, resulting in progressive ankle valgus. If left untreated, pathological hindfoot valgus and genu valgus are associated with an increased risk of early-onset degenerative osteoarthritis [7].

Pain is the predominant clinical symptom and may occur during activity or at rest, frequently resulting in reduced physical activity in affected children [8]. Clinical evaluation should extend beyond static assessment of coronal alignment to include dynamic examination of hindfoot valgus, such as the heel-rise test and the Jack test. Diagnostic workup must integrate detailed patient history and physical examination, while standing whole-leg radiographs remain the imaging modality of choice for deformity analysis.

Surgical correction is indicated in symptomatic children with persistent deformities, an intermalleolar distance exceeding 10 cm, progressive deformities, patellofemoral instability, gait disturbances, or significant deviation of the mechanical axis (MAD) from the knee center [1,6,9]. Hemiepiphysiodesis (HED) is a well-established technique for growth modulation in skeletally immature patients, temporarily inhibiting physeal growth on one side to achieve gradual correction. First described by Phemister in 1933 [10], this approach requires open physes with sufficient remaining growth potential. Preoperative planning relies on standing whole-leg radiographs to assess key angular parameters, including the anatomical femorotibial angle (aFTA), the anatomical lateral distal femoral angle (aLDFA), the medial proximal tibial angle (MPTA), and the lateral distal tibial angle (LDTA) [11,12]. Based on deformity pattern, treatment may involve monoarticular HED (targeting either the ankle or the knee) or biarticular HED (addressing both the ankle and the knee) [11]. With temporary guided growth techniques, continued physeal activity on the non-instrumented side enables gradual correction of the joint line. The tension plate technique, introduced in 2005, utilizes a non-locking two-hole plate bridging the physis and has demonstrated reliable clinical and radiographic outcomes [1,3,6]. The rate and extent of correction depend on the patient’s age and individual growth velocity [6]. In biarticular growth modulation, differing growth rates must be considered, as approximately 71% of femoral growth occurs at the distal femoral epiphysis and 57% of tibial growth at the proximal tibial physis [13]. Consequently, predicting the timing of correction and the optimal implant removal remains challenging, even when radiographic physiological angles are achieved [1,5,6]. Close clinical and radiographic follow-up is therefore crucial to avoid overcorrection or undercorrection [1]. At the knee, slight overcorrection of the mechanical axis (Mikulicz line) may be acceptable in the presence of open physes to mitigate potential rebound effects [1].

While the efficacy and safety of monoarticular growth modulation at either the knee or ankle are well established over recent decades, data on simultaneous ipsilateral correction of both joints remain limited [1,6]. To the best of our knowledge, no previous study has specifically evaluated concomitant ipsilateral biarticular growth modulation in this context [2,8]. Therefore, the aim of this case series was to assess the feasibility, safety and short-term radiographic outcomes of simultaneous ipsilateral biarticular growth modulation using hemiepiphysiodesis (HED) in children with concomitant knee and ankle valgus deformities (CKAVD). Furthermore, the findings are interpreted in the context of existing literature on monoarticular HED, with any comparisons considered exploratory and hypothesis-generating.

## 2. Materials and Methods

### 2.1. Ethical Approval and Consent

This study was approved by the Institutional Ethics Committee of the Medical Faculty at Leipzig University (approval number: 241/23-ek; dated 1 August 2023) and conducted in accordance with the principles of the Declaration of Helsinki and the guidelines for Good Clinical Practice. Written informed consent for the use of anonymized patient data was obtained from the parents or legal guardians, and informed consent for surgical intervention was obtained from all patients.

### 2.2. Subjects

**Figure 1:**
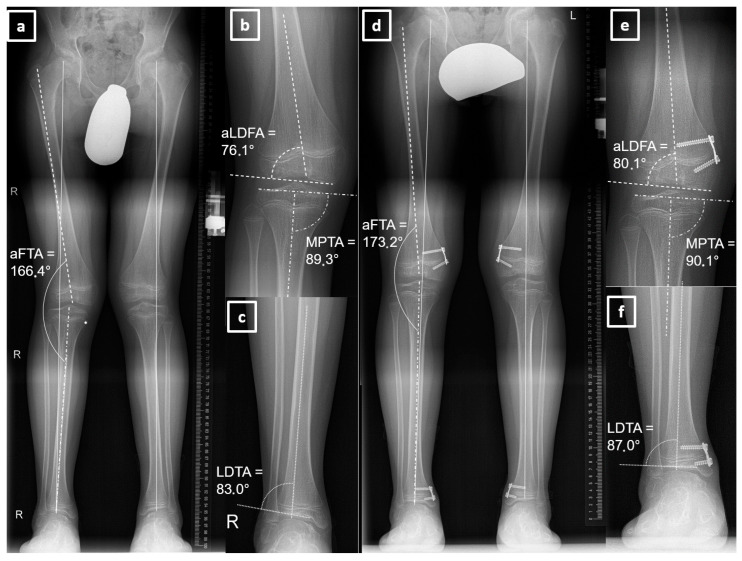
(**a**) Whole-leg radiograph of an 11-year-11-month-old boy with bilateral genua valga and hindfoot valgus, showing pathological distal femoral and distal tibial angles. (**b**) Close-up view of the right knee. (**c**) Close-up view of the right ankle. (**d**) Whole-leg radiograph of the same boy at age 12 years and 7 months, following 8 months of growth modulation with bilateral hemiepiphysiodesis at the distal femur and tibia. (**e**) Close-up view of the corrected right knee. (**f**) Close-up view of the corrected right ankle. aFTA—anatomical femorotibial angle; aLDFA—anatomical lateral distal femoral angle; MPTA—medial proximal tibial angle; LDTA—lateral distal tibial angle.

Inclusion criteria comprised skeletally immature patients (age < 18 years) requiring simultaneous ipsilateral growth modulation of the knee and ankle with open physes of the femur, tibia, and fibula. None of the patients had undergone previous surgery on the knee or ankle. Exclusion criteria included isolated monoarticular HED and incomplete radiographic or clinical data. The study period extended from implantation to a minimum of two years after complete hardware removal at both joints. Staged hardware removal did not constitute an exclusion criterion. An open growth plate was not a contraindication for hardware removal. Two-stage hardware removal did not constitute an exclusion criterion. The modulation period defined the period between implantation and the complete removal of the implants in both joints. Patients presenting with clinically evident hindfoot and knee valgus and an intermalleolar distance >10 cm underwent standardized standing long-leg radiographs, which served both diagnostic and progression assessment purposes [1]. Deformity analysis included measurement of the lateral distal tibial angle (reference range LDTA: 84–93°), anatomic femorotibial angle (reference range aFTA: 173–175°), anatomic lateral distal femoral angle (reference range aLDFA: 79–83°), and medial proximal tibial angle (reference range MPTA: 85–90°) to guide correction strategy (Figure 1) [1,10,11].

For patient-level analysis, the limb exhibiting the greatest coronal plane deviation, as determined by the FTA, was defined as the index extremity and included in the primary analysis, while results of the limb-level analysis are presented in Supplementary Table S1.

### 2.3. Intervention

All procedures were performed under general anesthesia using the standardized technique described by Stevens [3]. Temporary hemiepiphysiodesis was achieved with an approved tension-band-plate system (eight-Plate™, Orthofix, McKinney, TX, USA, or the EPI-Plate, Königsee Implantate, Allendorf, Germany) consisting of a low-profile plate and two cannulated, non-locking cancellous bone screws. For correction at the knee, a medial approach was used. After sharp dissection down to the periosteum, the tension plate was positioned along the mid-sagittal plane under biplanar fluoroscopic guidance. The tension plate was positioned along the longitudinal axis and preliminarily fixed with two K-wires through the screw holes. Following fluoroscopic confirmation, definitive fixation was achieved using two cannulated, non-locking cancellous bone screws. A 12 mm plate was used for the proximal tibia and a 16 mm plate for the distal femur. At the ankle, a medial approach was employed. After exposure of the periosteum, the implant (12 mm eight-Plate™ or EPI-Plate) was positioned along the longitudinal axis, and a K-wire was inserted through the distal hole of the implant under fluoroscopic guidance. Definitive fixation was also achieved using cannulated non-locking cancellous bone screws. Postoperatively, all patients were permitted immediate full weight-bearing and unrestricted range of motion.

### 2.4. Statistical Analysis

Statistical analysis was conducted using IBM SPSS Statistics (Version 25, IBM Corp, Armonk, NY, USA). Pre- and postoperative changes in continuous variables were analysed using paired-sample *t*-tests. Given the limited sample size, between-group comparisons were performed using the Mann–Whitney U test for continuous variables, and Fisher’s exact test for categorical variables. Data distribution was assessed for normality using the Shapiro–Wilk test. A *p*-value ≤ 0.05 was considered statistically significant. The intraclass correlation coefficient (ICC) was used to assess interrater reliability [14].

## 3. Results

### 3.1. Patients and Clinical Parameters

A total of 21 children were included in the study, of whom 17 underwent bilateral biarticular HED, while 4 were treated unilaterally by biarticular growth modulation. Among these patients, 14 right and 7 left lower extremities were designated as the index limb for analysis (Table 1).

All children underwent modulation at the medial distal tibia. In addition, modulation was performed in 13 children (62%) at the medial distal femur, in seven children (33%) at the proximal medial tibia, and in one child (5%) at both the medial distal femur and proximal tibia (Table 2).

Five children (24%) had idiopathic deformities with no underlying disease and seven children (33%) were obese. Secondary causes were observed in 9 patients (43%), including 4 cases of multiple hereditary exostoses (MHE) and 1 case each of clubfoot, Dravet syndrome, radiotherapy, mucopolysaccharidosis, and cerebellar astrocytoma.

Fourteen children (67%) were male, and 7 (33%) were female. The mean age at the initiation of HED was 11.5 years (range: 9.3–14.0; SD 1.1). Males were older at initiation (11.9 years, SD 1.0) compared to females (10.8 years, SD 0.9; *p* < 0.05). The average age at total hardware removal was 12.7 years (range: 11.2–14.9; SD 1.0). Females were younger at the time of hardware removal compared to males (*p* < 0.05; Table 1).

The mean modulation period was 14.3 months (SD 4.3 months), with no sex-based differences (*p* = 0.71). In 6 children (29%), a two-stage termination of HED was necessary, with hardware removal at the knee first, on average, 7 months before the ankle.

The mean height at the initiation of HED was 154.5 cm (SD 14.7 cm). Over the modulation period, the mean height increase was 7.0 cm (SD 4.1), with no difference between sexes (*p* = 0.86) or between idiopathic and secondary causes (*p* = 0.72).

Detailed patient characteristics and outcomes are summarized in Table 1.

### 3.2. Interobserver Reliability

The measurements were performed by two experienced orthopedic surgeons. The intraclass correlation coefficient (ICC) was high to very high for all parameters (ICC > 0.75) [14]. Detailed correlation levels are provided in Table 3.

### 3.3. Radiological Outcomes

The anatomical femorotibial angle (aFTA) improved significantly, with a mean increase of 6.9° (SD 2.6°; *p* < 0.05), rising from 168.0° to 175.0°. The mean rate of correction (ROC) for the FTA was calculated at 0.46° per month (SD 0.24°/month).

The lateral distal tibial angle (LDTA) also showed a substantial improvement, with a mean increase of 7.1° (SD 3.0°; *p* < 0.05). The mean ROC for the LDTA was 0.51° per month (SD 0.22°/month).

The lateral distal femoral angle (aLDFA) improved by a mean of 6.7° (SD 2.7°; *p* < 0.05), increasing from 76.4° to 83.1°. This angle was modulated in 13 children, with a mean ROC of 0.34° per month (SD 0.33°/month).

The medial proximal tibial angle (MPTA) showed a mean correction of 7.2° (SD 2.3°; *p* < 0.05), decreasing from 93.4° to 86.1°. Modulation was performed in 7 children, with a mean ROC of 0.46° per month (SD 0.2°/month).

No differences were observed between children with primary and secondary deformities in the magnitude of correction for aFTA, aLDFA, MPTA and LDTA (*p* > 0.05).

**Figure 2:**
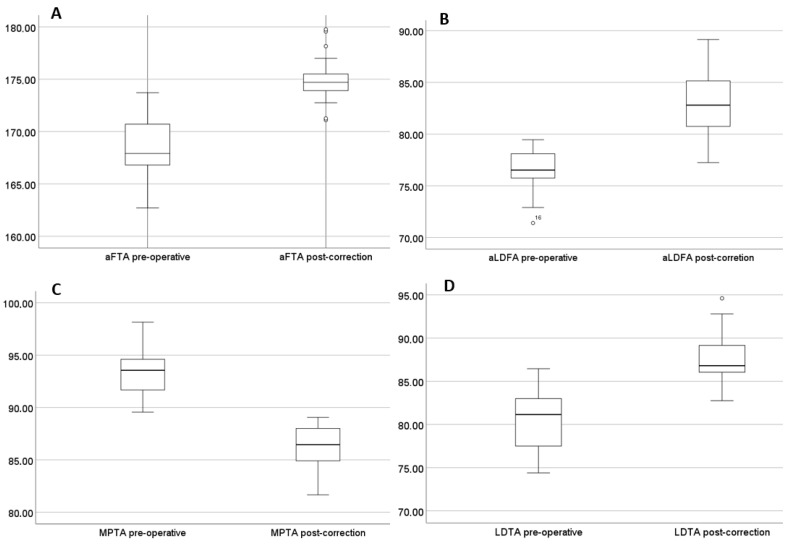
(**A**–**D**): Graph (box plot) of development in degree of (**A**) aFTA—anatomical femorotibial angle; (**B**) aLDFA—anatomical lateral distal femoral angle; (**C**) MPTA—medial proximal tibial angle; (**D**) LDTA—lateral distal tibial angle pre-operative and post-correction as a function of localization.

None of the modulated ankles were overcorrected. A detailed overview and gender comparison of the radiological parameters and their improvement during the growth modulation period are provided in Table 2 and Figure 2.

Supplementary Table S1 provides an overview of the statistical analyses performed at the limb level, in which each extremity (N = 38), regardless of whether one or both limbs (N = 17) of a given patient were treated, was considered as an individual analytical unit.

Ipsilateral biarticular growth modulation resulted in a significant reduction in intermalleolar distance, with an average decrease of 10 cm, especially in children with growth modulation of both lower extremities (SD 4.5; *p* < 0.05; Supplementary Table S1).

### 3.4. Complications

During the study period, one patient experienced a relapse of knee valgus following hardware removal. After hardware removal at the ankle, a monoarticular revision of the knee joint with a repeat HED procedure was required. However, no clinical recurrence of ankle valgus deformity was observed. Additionally, there were no complications related to wound healing or the hardware. No complications were observed during the treatment course in any child who underwent staged hardware removal.

## 4. Discussion

In this retrospective single-center case series of 21 lower extremities in 21 consecutive children with concomitant knee and ankle valgus deformities (CKAVD), simultaneous ipsilateral biarticular hemiepiphysiodesis resulted in substantial radiographic improvement of ankle and knee valgus alignment. The mean correction of the LDTA was 7°, accompanied by a reduction in intermalleolar distance of 10 cm. Two-stage hardware removal was required in 6 patients (29%).

Monoarticular temporary hemiepiphysiodesis (HED) is well established for the treatment of coronal plane deformities of the knee or ankle and has been consistently supported by reliable evidence [1,2,3,4,5,6,9,15,16]. In contrast, evidence on simultaneous biarticular growth modulation remains limited. CKAVD appears to be uncommon, and prior reports are largely restricted to small case descriptions [2,8]. The present study demonstrates the feasibility and short-term effectiveness of simultaneous ipsilateral biarticular HED, with most children achieving correction toward physiological alignment at both joints. The present findings suggest that ipsilateral biarticular HED achieves a magnitude of correction within the range reported for monoarticular HED; however, in the absence of a control group, the conclusions regarding comparative efficacy remain indirect and hypothesis-generating. In this cohort, mean improvements were 6.9° in the anatomical femorotibial angle (aFTA) and 7.1° in the lateral distal tibial angle (LDTA), consistent with previously published ranges of 7–10° for aFTA and 6–13° for LDTA following monoarticular HED [2,4,6,16,17,18,19,20]. Accurate deformity analysis remains essential, particularly in multilevel pathologies such as CKAVD. Treatment planning must consider individual growth potential, underlying etiology, and the anatomical location of the deformity [4,6,8,16]. Moreover, open physes and a sufficient remaining growth potential of at least one year at both joints are essential prerequisites for hemiepiphysiodesis [3,5,6]. However, these timing requirements pose a particular challenge in the management of CKAVD. In the present study, simultaneous biarticular HED was associated with a mean modulation period of 14.3 months, which is consistent with reported durations ranging from 9.5 to 18 months for the knee and 14 to 36 months for the ankle [1,2,3,6,8,16,17,18]. Similarly, the observed rate of correction at both joints was in line with published data, with values typically reported between 0.5 and 0.8° per month for the knee and 0.36 to 0.6° per month for the ankle [2,3,5,15,19]. Notably, younger patients have been shown to exhibit greater correction potential, which should be taken into account during treatment planning and monitoring of growth modulation [6].

The present analysis indicates that biarticular HED yields promising results, while also highlighting areas for further optimization in the context of existing literature. At the time of complete hardware removal, the mean LDTA for all patients was 87.6°, corresponding to the lower end of the physiological reference range, whereas the aFTA demonstrated correction toward the upper reference range [11].

To prevent complications such as overcorrection of the knee, a two-stage termination procedure was employed in six children (29%), with initial hardware removal at the knee followed by removal at the ankle. A staged hardware removal strategy may facilitate greater correction at the ankle, potentially allowing the LDTA to approach the ideal value of 90°, particularly in the context of longer modulation periods as reported in the literature [4,6,8,19]. In the present series, single-stage hardware removal was frequently preferred once satisfactory knee and ankle alignment had been achieved, primarily to avoid an additional surgical procedure requiring anesthesia and its associated risks. Although no increased complication rate was observed with staged removal, this finding should be interpreted with caution in light of the limited sample size. Future research should aim to develop an algorithm for the optimal treatment course for multi-articular coronal deformities, ideally in a multicenter setting.

The observed complication rate was 5%. One child experienced a relapse of bilateral valgus deformity in the knee. These findings are consistent with the complication rates reported for monoarticular HED procedures, reaffirming the safety profile of biarticular interventions [19,20].

The retrospective design of this study, its single-center setting, and the heterogeneity of the patient cohort are notable limitations. The relatively small sample size may represent the primary limitation. The etiological heterogeneity of this case series reflects the real-world spectrum of CKAVD encountered at a university orthopedic center. The majority of patients presented with idiopathic or obesity-related deformities (n = 12), which are characterized by comparable physeal physiology and correction kinetics. In contrast, the remaining patients exhibited underlying conditions affecting bone metabolism or physeal integrity, including MHE, mucopolysaccharidosis, and radiation-induced deformity, where correction behavior may differ substantially. Although no significant difference in intermalleolar distance correction was observed between idiopathic and secondary etiologies, the study is underpowered for meaningful subgroup inference. Therefore, the pooled quantitative outcomes should be interpreted with caution and not generalized across all etiologies. Future prospective studies with stratified analyses are warranted to better characterize etiology-specific correction patterns and clinical outcomes. A further limitation is the analytical approach, although the primary analysis was conducted at the patient level using an index limb, supplementary limb-level analyses treated extremities as independent observations, potentially introducing bias due to within-subject correlation in bilateral cases. However, the sample size and statistical analysis are comparable to those of similar studies reported in the literature, especially for the ankle joint [1,6,8,17]. In the authors’ opinion, the use of two different plate systems can be considered a minor limitation, as both have been demonstrated to be effective and functionally equivalent for guided growth using tension-band plating [1,8].

## 5. Conclusions

Comprehensive evaluation of all major joints is essential in the management of coronal plane deformities, particularly in multi-level deformities, such as CKAVD. For these children, simultaneous ipsilateral biarticular hemiepiphysiodesis (HED) may be considered as a feasible treatment approach, demonstrating promising short-term outcomes and a low observed complication rate. However, these findings should be interpreted with caution in patients with underlying conditions affecting physeal integrity, such as skeletal dysplasia or metabolic bone disease, for whom etiology-specific prospective data are lacking. Careful patient selection, meticulous surgical planning, and close follow-up are critical. The possibility of a two-stage treatment termination should be transparently discussed with patients and their guardians before initiating HED. Given the study design, conclusions are limited to feasibility and short-term outcomes, and further prospective, controlled studies are required to define optimal treatment strategies.

## Figures and Tables

**Table 1:** Overview of the main patient characteristics during the study period. *p*-values represent comparisons between female and male patients using the Mann–Whitney U test.

Parameter	All	Female	Male	*p*-Value
Patients (*n*)	21	7	14	
unilateral [*n* (%)] bilateral [*n* (%)]	4 (19%) 17 (81%)	2 5	2 12	0.26
underlying cause primary secondary	12 9	3 4	9 5	0.32
Body height (SD) in cm before growth modulation Range	154 (14.7) 122–175	149.0 (16.5) 122–174	157.1 (13.6) 130–175	0.25
Increase in body height (SD) in cm Range	7 (4.08) 1.0–18.0	7.0 (4.55) 1.0–16.0	7.1 (4.01) 2.0–18.0	0.86
Mean age at implantation (SD) in years Range	11.5 (1.1) 9.3–14.0	10.8 (0.95) 9.3–12.0	11.9 (0.98) 10.8–14.0	<0.05
Modulation period (SD) in months Range	14.3 (4.3) 8.1–24.2	13.8 (4.7) 9.7–23.3	14.5 (4.2) 8.1–24.2	0.71
Mean age at termination (SD) in years Range	12.7 (1.0) 11.2–14.9	11.92 (0.74) 11.2–13.2	13.09 (0.89) 12.0–14.9	<0.05

**Table 2:** Detailed overview and gender comparison of radiological parameters and their progression during the growth modulation period. *p*-value ^1^ (pre-operative vs. post-correction): paired-samples *t*-tests. *p*-value ^2^ (sex comparison): Mann–Whitney U test. aFTA—anatomical femorotibial angle; aLDFA—anatomical lateral distal femoral angle; MPTA—medial proximal tibial angle; LDTA—lateral distal tibial angle; SD—standard deviation. ROC—rate of correction.

Parameter	All	*p*-Value ^1^	Female	Male	*p*-Value ^2^
Patients (*n*)	21		7	14	
Legs (*n*) Left Right	21 7 14		7 1 6	14 6 8	0.21
aFTA (*n* = 21) in degrees pre-operative (SD) post-correction (SD) Magnitude of correction ROC in degree/month (SD)	168.0 (2.92) 175.0 (2.29) 6.9 (2.6) 0.46 (0.24)	<0.05	166.4 (2.2) 174.5 (0.8) 6.7 (1.3) 0.52 (0.15)	168.9 (2.9) 175.2 (2.7) 5.6 (3.2) 0.43 (0.28)	0.06 0.51 0.28 0.17
LDTA (*n* = 21) in degrees pre-operative (SD) post-correction (SD) Magnitude of correction (SD) ROC in degree/month (SD)	80.4 (3.2) 87.6 (2.8) 7.1 (3.0) 0.51 (0.22)	<0.05	79.9 (3.4) 87.3 (3.3) 7.4 (3.5) 0.53 (0.35)	80.7 (3.3) 87.7 (2.7) 7.0 (2.9) 0.51 (0.21)	0.63 0.74 0.82 0.81
aLDFA (*n* = 13) in degree pre-operative (SD) post-correction (SD) Magnitude of correction (SD) ROC in degree/month (SD)	76.4 (2.3) 83.1 (3.4) 6.7 (2.7) 0.34 (0.33)	<0.05	75.6 (2.9) 83.1 (4.1) 7.5 (2.1) 0.44 (0.35)	76.8 (2.0) 81.7 (3.6) 4.9 (2.7) 0.30 (0.32)	0.41 0.43 0.15 0.37
MPTA (*n* = 7) in degree pre-operative (SD) post-correction (SD) Magnitude of correction (SD) ROC in degree/month (SD)	93.4 (2.6) 86.1 (2.6) 7.2 (2.3) 0.46 (0.2)	<0.05	95.8 (2.1) 88.2 (1.0) 7.6 (1.3) 0.53 (0.15)	92.0 (1.8) 85.0 (2.2) 7.0 (2.8) 0.42 (0.24)	0.04 0.06 0.74 0.52

**Table 3:** Intraclass correlation coefficient (ICC) representing interrater reliability, with ICC > 0.75 indicating a high level of agreement [14]. aFTA—anatomical femorotibial angle; aLDFA—anatomical lateral distal femoral angle; MPTA—medial proximal tibial angle; LDTA—lateral distal tibial angle.

	Pre-Operative				Post-Correction			
Parameter	aFTA	aLDFA	MPTA	LDTA	aFTA	aLDFA	MPTA	LDTA
ICC	0.967	0.946	0.962	0.885	0.970	0.884	0.764	0.864

## Data Availability

The data presented in this study are available upon request from the corresponding author. The data are not publicly available due to privacy and ethical reasons.

## Supplementary Materials

The following supporting information can be downloaded at: https://www.mdpi.com/article/10.3390/children13050675/s1, Table S1 provides a detailed overview and gender-specific comparison of radiological parameters and their trends during the growth modulation phase for all lower extremities, regardless of whether bilateral or unilateral growth modulation was present.

## Abbreviations

The following abbreviations are used in this manuscript:

aFTA	anatomic femorotibial angle
aLDFA	anatomic lateral distal femoral angle
MPTA	medial proximal tibial angle
CKAVD	concomitant knee and ankle valgus deformities
HED	hemiepiphysiodesis
LDTA	lateral distal tibial angle
ROC	rate of correction
