# Supplementary material for: Simultaneous Biarticular Growth Modulation for Ipsilateral Concomitant Valgus Deformities of the Knee and Ankle: Short-Term Results of a Case Series
Source: Children (Basel). 2026 May 14;13(5):675. doi: 10.3390/children13050675 (PMC13204489; doi:10.3390/children13050675)
Supplement: Supplementary file 1 [file children-13-00675-s001.zip › children-4232891-supplementary.pdf]

Supplementary Table S1. Detailed overview and gender comparison of radiological parameters and their progression during the growth modulation period of all lower extremities including bilateral growth modulation. aFTA – anatomical femorotibial angle; aLDFA – anatomical lateral distal femoral angle; MPTA – medial proximal tibial angle; LDTA – lateral distal tibial angle; SD – standard deviation.

Parameter	All	female	male	p
Patients (n)	21	7	14	
Legs (n)	38	12	26	
Left	18	5	13	0.734
right	20	7	13	
Correction level (at knee)				
Femoral	23	7	16	
Tibial	12	5	7	
Femoral + tibial	3	0	3	
**aFTA** (N = 38) in degree				
pre-operative (SD; range)	168.4 (3.36; 162.7 – 175.6)	166.5 (2.4)	169.3 (3.4)	**0.016**
post-correction (SD; range)	175.4 (2.08; 171.1 – 179.8)	174.7 (0.9)	175.7 (2.4)	0.143
Magnitude of correction (SD; range)	7.0 (2.9; 1.0 – 12.2)	8.2 (1.8)	6.5 (3.2)	0.100
ROC in degree/month (SD, range)	0.5 (0.2; 0.1 – 0.9)	0.52 (0.11)	0.44 (0.28)	0.332
**LDTA** (N = 38) in degree				
pre-operative (SD; range)	80.4 (3.3; 73.1 – 86.5)	79.6 (3.6)	80.8 (3.1)	0.326
post-correction (SD; range)	87.6 (2.7; 82.6 – 94.6)	87.2 (2.5)	87.7 (2.8)	0.608
Magnitude of correction (SD; range)	7.2 (2.3 – 14.6)	7.6 (3.8)	7.0 (3.4)	0.602
ROC in degree/month (SD; range)	0.5 (0.2; 0.1 – 1.0)	0.53 (0.22)	0.52 (0.26)	0.862
**aLDFA** (N=26) in degree				
pre-operative (SD; range)	76.9 (2.2; 71.4 – 81.50)	76.3 (2.5)	77.2 (2.1)	0.402
post-correction (SD; range)	82.6 (3.4; 75.8 – 89.2)	83.6 (3.2)	82.2 (3.4)	0.224
Magnitude of correction (SD; range)	5,7 (2.7; 1.4 – 10.9)	7.3 (2.2)	5.1 (2.8)	0.111
ROC in degree/month (SD; range)	0.4 (0.3; 0.2 – 0.8)	0.42 (0.35)	0.32 (0.32)	0.423
**MPTA** (N=15) in degree				
pre-operative (SD; range)	93.6 (4.3; 89.1 - 105.4)	98.0 (4.4)	91.4 (1.7)	**0.001**
post-correction (SD; range)	86.5 (81.7 – 91.1)	89.1 (1.5)	85.1 (1.9)	**0.001**
Magnitude of correction (SD; range)	7.1 (3.0; 2.9 – 14.3)	8.9 (3.2)	6.3 (2.6)	0.103
ROC in degree/month (SD; range)	0.4 (0.2; 0.2 – 0.8)	0.54 (0.11)	0.37 (0.21)	0.121
